# Selective and self-validating breath-level detection of hydrogen sulfide in humid air by gold nanoparticle-functionalized nanotube arrays

**DOI:** 10.1007/s12274-021-3771-7

**Published:** 2021-09-02

**Authors:** Luis Antonio Panes-Ruiz, Leif Riemenschneider, Mohamad Moner Al Chawa, Markus Löffler, Bernd Rellinghaus, Ronald Tetzlaff, Viktor Bezugly, Bergoi Ibarlucea, Gianaurelio Cuniberti

**Affiliations:** 1 Institute for Materials Science, Max Bergmann Center of Biomaterials, Technische Universität Dresden, Dresden, 01062 Germany; 2 Institute of Circuits and Systems, Technische Universität Dresden, Dresden, 01062 Germany; 3 Dresden Center for Nanoanalysis (DCN), Center for Advancing Electronics Dresden (cfaed), Technische Universität Dresden, Dresden, 01062 Germany; 4 Life Science Incubator Sachsen GmbH & Co. KG, Dresden, 01307 Germany; 5 Center for Advancing Electronics Dresden (cfaed), Technische Universität Dresden, Dresden, 01062 Germany

**Keywords:** chemiresistive gas sensors, semiconducting carbon nanotubes, gold nanoparticles, hydrogen sulfide detection, chemiresistor mathematical model

## Abstract

We demonstrate the selective detection of hydrogen sulfide at breath concentration levels under humid airflow, using a self-validating 64-channel sensor array based on semiconducting single-walled carbon nanotubes (sc-SWCNTs). The reproducible sensor fabrication process is based on a multiplexed and controlled dielectrophoretic deposition of sc-SWCNTs. The sensing area is functionalized with gold nanoparticles to address the detection at room temperature by exploiting the affinity between gold and sulfur atoms of the gas. Sensing devices functionalized with an optimized distribution of nanoparticles show a sensitivity of 0.122%/part per billion (ppb) and a calculated limit of detection (LOD) of 3 ppb. Beyond the self-validation, our sensors show increased stability and higher response levels compared to some commercially available electrochemical sensors. The cross-sensitivity to breath gases NH_3_ and NO is addressed demonstrating the high selectivity to H_2_S. Finally, mathematical models of sensors’ electrical characteristics and sensing responses are developed to enhance the differentiation capabilities of the platform to be used in breath analysis applications.

**Electronic Supplementary Material:**

Supplementary material (details on the dielectrophoretic deposition, AuNP functionalization optimization, full range of experimental and model H_2_S sensing response up to 820 ppb, and sensing response to NO gas) is available in the online version of this article at 10.1007/s12274-021-3771-7.

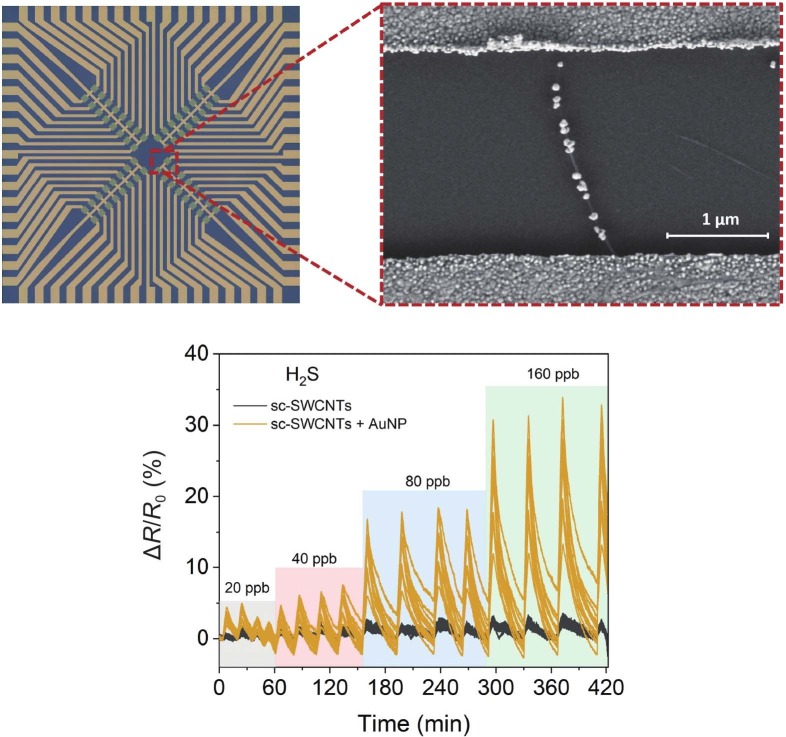

## KEYWORDS

chemiresistive gas sensors, semiconducting carbon nanotubes, gold nanoparticles, hydrogen sulfide detection, chemiresistor mathematical model

## 1 Introduction

During the last decade, the potential of exhaled breath analysis as a non-invasive and cost-effective alternative for precise disease identification and health monitoring has been intensively considered [1–6]. Human breath contains over 3,500 components [7, 8] including $N_2$, $O_2$, $CO_2$, and volatile organic compounds (VOCs), presenting a great challenge for sensing technologies in terms of sensitivity, selectivity, and response time. To this end, several gas sensing technologies for breath analysis have been intensively investigated over the past few years, exploring the increased sensitivity and selectivity of nanostructured materials [9–14]. Human exhaled breath is mainly composed of $N_2$ (78.04%), $O_2$ (16%), $CO_2$ (4%–5%), inert gases (0.9%), and water vapor [15]. However, many other gases and VOCs are present as well in much smaller concentrations. Variations in their levels or the appearance of new ones can indicate a certain disease or condition. While the acetone odor was already a known marker of diabetic coma since more than a century ago [16], the development of analytical methods in modern science, and especially with highly sensitive sensing devices, determined several of the compounds that could be attributed to specific health conditions. Variations in isobutane, toluene, or ethanol among others, for example, can be used as markers of lung cancer [17], while the pulmonary infections by pathogens can be identified through the fingerprinting of bacteria-specific VOCs [18]. $H_2S$ in particular is a naturally exhaled breath gas with origin in the sulfate-reducing bacteria [19], which can be monitored at low part per billion (ppb) levels as marker of small intestinal bacterial overgrowth and irritable bowel syndrome [19, 20]. The odor threshold for such gas is around 3 ppb [21], making it possible to perform a pre-diagnosis via a simple smelling step, identifying halitosis [22]. However, a more reliable quantification and clinical diagnostic procedure should be carried out afterwards.

Two of the main bottlenecks to achieve a precise diagnosis are

TSINGHUA UNIVERSITY PRESS Springer the selectivity issues of gas sensors and the sensitivity required for such low levels. Additional features in terms of stability, reproducibility, and fast response time are also critical characteristics that, together with the miniaturization possibilities offered by micro- and nanofabrication technologies, can ultimately lead to the development of robust diagnostic devices with a portable and comfortable maneuver.

In this context, the implementation of nanomaterials, and especially 1D materials such as carbon nanotubes (CNTs) [23] or silicon nanowires [24, 25] has boosted the sensitivity of gas sensors in low power consumption formats. Ever since they were first used as chemical sensors 20 years ago [26], CNTs have been extensively investigated as sensor transducers for the sensitive and selective detection of gas molecules. In particular, semiconducting single-walled CNTs (sc-SWCNTs) have been demonstrated to be the most promising alternative to be used as a starting point to address specific sensing applications by further chemical or physical modification [27]. Their reduced length in the range of a few micrometers and their atom-thickness make them ideal for sensing small external perturbations affecting their electrical properties.

To date, several works have demonstrated CNT-based devices capable of reaching ppb detection levels of VOCs such as isopropanol, acetone, and isoprene [28], or gases such as nitric oxide [29] or ammonia [30]. In relation to $H_2S$, various chemical modifications of the CNT surface can provide the necessary selectivity. For example, the surface immobilization of 2,2,6,6-tetramethylpiperidine-1-oxyl (TEMPO) was shown to catalyze redox reactions of the target gas and water vapors [31], leading to an electron donation process that resulted in a resistance increase. However, the sensitivity demonstrated using this modification approach was not enough for breath analysis applications. An alternative approach with the capability of ppb detection can be found in the CNT decoration with gold nanoparticles (AuNPs) following a simple electrodeposition process. Moreover, due to the affinity between gold and sulfur atoms, the sensing could be achieved selectively [32].

In this work, we present the selective detection of low ppb concentrations of $H_2S$ in humid air and at room temperature by AuNP-functionalized sc-SWCNTs-based chemiresistive sensors. We demonstrate the reproducible and wafer-scale compatible fabrication of small footprint 2.4 cm × 2.4 cm devices consisting of 64 gas sensors each, based on a controlled dielectrophoretic deposition of the nanotubes followed by a potentiostatic electrodeposition of AuNP. The consistent behavior of the sensor array works as a self-validating mechanism, which can be combined with an unmodified array on the same chip for complementary measurements of ammonia. The sensing response of both functionalized and non-functionalized sensors to low ppb concentrations of $H_2S$, $NH_3$, and NO was systematically studied under the same conditions and mathematically modeled toward the improvement of a predictive approach and enhanced target gas differentiation capability.

## 2 Results and discussion

### 2.1 Gas sensor fabrication

A standard photolithography process on 4” silicon wafers results in the production of 2.4 cm × 2.4 cm multichannel devices (Figs. 1(a)–1(c)). Each device consists of 64 individual sensors grouped in 4 quadrants of 16 sensors each with a common source/working electrode used for dielectrophoretic deposition of sc-SWCNTs (Fig. 1(d)), electrochemical deposition of AuNP (Fig. 1(e)), and the measurement of the output characteristics (Fig. 1(f)). A complete description can be found in the experimental section.

Dielectrophoresis (DEP) has been intensively studied as a reliable method for the fabrication of CNT-based devices [33–36]. The application of a non-uniform alternating current (AC) electric field creates a dielectrophoretic force that induces a dipole moment on CNTs, thus aligning the nanotubes in the direction of the electric field gradient between metal electrodes [37] (Fig. 1(d)). This technique presents several advantages compared to chemical vapor deposition (CVD) alignment [38, 39], such as room-temperature operation, and the absence of post-synthesis transferring processes. Moreover, its simplicity, good reproducibility, cost-effectiveness, and more importantly, the efficient individualization of CNTs between electrodes make DEP the most attractive alternative for the fabrication of CNT-based gas sensors compared to other solution processing methods like drop-casting [23, 30] and self-assembly monolayer (SAM) assisted deposition [40, 41]. The DEP deposition of sc-SWCNTs on 64-sensor devices results in the alignment of individual and small tube agglomerations between metal electrodes providing an increased active surface area for interaction with gas molecules (Fig. 1(g)). Moreover, a narrow distribution of sensors’ electrical resistance in the low kΩ rage is achieved which corresponds to the optimal resistance values proposed in our previous study [23]. It is important to notice that, even though the dielectrophoretic deposition process is stopped at 14 kΩ for all 64 sensors, the difference in sensors’ final resistance might come from the instability of the N-methyl-2-pyrrolidone (NMP)/sc-SWCNTs dispersion over the whole deposition process along with the presence of small unavoidable defects at the microelectrodes (Fig. S1 in the Electronic Supplementary Material (ESM)).

The optimization of AuNP functionalization described in the ESM demonstrates the capability of using a gold-containing electrolyte and a potentiostatic deposition to accurately tune the particle size and distribution by the modification of voltage and deposition times. The selected parameters of −0.4 V and 30 s lead to the optimal nucleation at the defect sites of sc-SWCNTs and nanotube-nanotube junctions which are more chemically reactive than the continuous lattice [42, 43]. The average particle diameter is 60 nm and the distance between particles on an individual nanotube agglomeration is at least 100 nm (Fig. 1(h)). Since the common electrode for each sensor group was used as a working electrode, the synthesis of AuNP was also observed at the electrode edges affecting the already existing Schottky junctions between sc-SWCNTs and metallic electrodes.

Electrical characterizations before and after AuNP deposition show a decrease in resistance for all sensors. For non-functionalized devices, this result might be attributed to the continuous evaporation of residual NMP after device fabrication. On the other hand, for AuNP-functionalized devices, the deposition of particles on the sc-SWCNTs walls results in a further resistance drop of around 30% (Fig. 1(i)), which corresponds to the small charge transfer from the nanotubes to the AuNP as calculated in previous reports [44, 45]. Moreover, the $I$–$V$ curves (Fig. 1(f)) of non-functionalized sensors show an expected semiconducting S-shape indicating Schottky contacts between gold electrodes and sc-SWCNTs, whereas higher source-drain current $I_{SD}$ values were measured for AuNP-functionalized devices because of the mentioned charge transfer.

### 2.2 Gas sensing performance

First, we assess the effect of AuNP functionalization on the sensing response to low ppb concentrations of $H_2S$ and $NH_3$ gas. For this, the sensors were exposed to 4 continuous cycles of 20, 40, 80, and 160 ppb of $H_2S$ and $NH_3$ using humid air (25% at 27 °C) as the

**Figure 1** Fabrication and characterization of gas sensing devices. (a) Photographic image of microelectrodes fabricated by UV lithography and metal deposition processes on a 4” $Si/SiO_2$ wafer. (b) Optical image of the multichannel device comprising 64 individual gas sensors distributed in four groups of 16, each one sharing a common source/working electrode. (c) Individual gas sensor with an IDE area of 500 μm × 500 μm and a 2-μm gap between electrodes. Schematics of (d) the controlled dielectrophoretic deposition of sc-SWCNTs and (e) AuNP electrodeposition using a three-electrode configuration and 1 mM $KAuCl_4$ in 100 mM KCl as an electrolyte. (f) Output characteristics (*I*–*V* curves) of AuNP-functionalized and non-functionalized sensors. SEM images of aligned sc-SWCNTs between electrodes after (g) DEP deposition and (h) after AuNP functionalization. (i) Resistance change (%) of functionalized and non-functionalized sensors after AuNP deposition process with respect to the resistance values after DEP deposition.

carrier medium. Each cycle comprised 5 min exposure to the analyte gas and recovery under humid air. The recovery time was different for each concentration. The change of the current at a fixed source-drain voltage ($V_{SD}$) of 100 mV was measured for the 64 sensors of a single chip using a custom-built multiplexing platform, and the sensor response was calculated as the relative resistance change using Eq. (1) where $\Delta R_{(t)}$ is the difference of resistance during ($R_t$) and before ($R_0$) analyte gas exposure [46]

$$\text{Response}\,(S)\,(\%) = \frac{\Delta R_{(t)}}{R_0} \times 100 \qquad (1)$$

Previous to analyte gas exposure, the sensors were stabilized in a continuous airflow of 6 L/min for about 2 h. After stabilization time, the temperature and relative humidity values were kept constant during analyte exposure experiments.

In general, AuNP-functionalized and non-functionalized sensors show an expected increase in resistance after exposure to $H_2S$ and $NH_3$ gases as can be seen in Fig. 2. The sensing response systematically increases by increasing the gas analyte concentration and is reproducible over the four continuous cycles with minimal variations demonstrating a robust reproducibility. The interaction of p-type sc-SWCNTs with such electron-donating gases results in the depletion of charge carriers (holes), thus increasing the electrical resistance of the system [26, 47]. Nevertheless, specific sensing mechanisms can be further discussed for each gas.

Regarding hydrogen sulfide, unmodified sc-SWCNTs exhibit a reduced sensitivity to all concentrations compared to AuNP-functionalized devices (Figs. 2(a) and 2(c)). This result comes from the weak binding energies between $H_2S$ and graphitic surfaces [48], indicating loose physisorption and charge transfer from the $H_2S$ molecules to the sc-SWCNTs channels. Therefore, Schottky barrier modulation occurring at the sc-SWCNTs/Au electrode interface is expected to be the major contribution to resistance change. A certain degree of adsorption of $H_2S$ to CNTs through hydrogen bonds, as reported elsewhere, cannot be discarded as well [49]. On the contrary, the enhanced sensitivity of AuNP-functionalized sensors originates from the high chemical affinity of sulfur and gold, producing a catalytic spillover effect on the particle surfaces at the AuNP/nanotube interface [50]. A sensing mechanism proposed by Mubeen et al. [32, 51] based on studies from Geng et al. [52] and Leavitt and Beebe [53] suggests that $H_2S$ is first chemisorbed on the AuNP surface and then decomposed into Au-SH or Au-S species which lower the particles’ work function. This effect at the so-called “nano-Schottky barriers” produces the electron donation from AuNP to sc-SWCNTs which increases the electrical resistance of the system. For our sensors, the optimized size (average particle diameter of 60 nm) and sparse distribution of AuNP on individualized sc-SWCNTs result in a remarkable average sensing response of 2.2%, 5%, 12%, and 21% for 20, 40, 80, and 160 ppb respectively at room temperature and relative humidity of ~ 25%. The response shows good linearity

**Figure 2** Sensing response ($\Delta R/R_0$) of AuNP-functionalized and non-functionalized sensors to 20, 40, 80, and 160 ppb of (a) $H_2S$ and (b) $NH_3$. Colored areas are displayed to indicate the time-frame of 4 cycles for each gas concentration. Average sensing response of 32 sensors for each (c) $H_2S$ and (d) $NH_3$ concentration respectively.

($R^2$ = 0.98) and sensitivity of 0.122%/ppb at low ppb range and slight saturation for higher concentrations as can be seen in Fig. S3 in the ESM. Moreover, the limit of detection (calculated using the 3α/slope definition) is 3 ppb, lower than other chemiresistive CNT-based sensors (Table 1), and comparable to previously reported AuNP-functionalized SWCNTs [32] as well as to the odor threshold.

Furthermore, a higher response and signal stability compared to commercially available electrochemical (EC) sensor ($H_2S$-B4, Alphasense, Essex, UK) in the low ppb range was observed. As can be seen in Fig. 3(a), the EC-sensor shows no noticeable detection to four continuous cycles of 20 ppb, whereas AuNP-functionalized devices exhibit reproducible results with a slight negative drift. In addition, the calculated signal-to-noise ratio outperforms the commercial sensor for all tested concentrations (Fig. 3(b)) allowing a clear distinction after only 60 s of exposure (Figs. 3(c) and 3(d)) demonstrating a faster response with the added value of the presence of multiple sensors running the measurements in parallel.

After $H_2S$ flow is stopped, the resistance returns to the initial baseline under humid airflow. It has been suggested that the removal of SH from gold surfaces by disproportionation is favored only at high temperatures [53]. However, we observe a complete recovery at room temperature which better fits the study reported by Jaffey and Madix [54], where SH disproportionation occurs at 320 K. We believe that efficient recovery is not only influenced by temperature but also by the effect of $O_2$ and $H_2O$. To test this hypothesis, the sensors were exposed to 60 ppb of $H_2S$ for 15 min using pure dry $N_2$ as the carrier gas at room temperature. As it can be seen in Fig. S4(a) in the ESM, the resistance does not decrease during recovery time, demonstrating the importance of air components for the efficient removal of SH at room temperature. It can also be noticed that the sensing response is significantly decreased in pure nitrogen atmosphere where an average response of only 2% is measured for longer exposure times (15 min) and higher concentrations (60 ppb). This suggests that $O_2$ and $H_2O$ are not only crucial for an efficient sensor recovery, but also play an important role in the sensing mechanism. To further investigate

**Table 1** Literature reported $H_2S$ gas sensors

| Material | $H_2S$ (ppm) | Response | Response time | Carrier gas | $T$ (°C) |
|---|---|---|---|---|---|
| SWCNTs/TEMPO [31] | 10 | 4.7% | ~ 5 min | Dry $N_2$ | Room $T$ |
| Au-catalyzed $SnO_2$ nanocolumn [62] | 5 | 99% | 500 s | Dry $N_2$ | 300 |
| MWCNTs/AuNP [63] | 1 | 1.3% | 10 min | Dry air | 150 |
| MWCNTs/Pt nanoclusters [64] | 1 | 5.5% | 10 min | Dry air | 200 |
| AgNP-doped graphene [65] | 0.1 | 10% | 6 min | Ar | Room $T$ |
| CNTs/Au-nano clusters [50] | 0.1 | 4.5% | 10 min | Dry air | 200 |
| COOH-SWCNTs/AuNP [32] | 0.02 | ~ 6% | ~ 5 min | Dry air | Room $T$ |
| COOH-SWCNTs/AuNP [51] | 0.002 | 3.75% | ~ 10 min | Dry air | Room $T$ |
| Polyaniline NW/AuNP [66] | $1 \times 10^{-4}$ | 20% | ~ 5 min | Dry air | Room $T$ |
| sc-SWCNTs/AuNP (this work) | 0.02 | 2.2% | 5 min | Humid air | Room $T$ |

**Figure 3** (a) Sensing response of AuNP-functionalized (yellow lines) and EC-sensor H2S-B4 (black line) to four continuous cycles of 20 ppb $H_2S$. (b) Signal-to-noise ratio for all tested concentrations calculated by dividing the average sensing response of 4 exposure cycles by the standard deviation of the noise signal. Sensing response of (c) AuNP-functionalized sensors and (d) EC-sensor to all $H_2S$ concentrations after 60 s exposure. Colored areas represent the standard deviation of AuNP-functionalized sensors responses and EC signal noise, respectively.

this claim, a systematic exposure experiment to 100 ppb of $H_2S$ at 5% and 50% relative humidity was performed. As it can be seen in Fig. S5 in the ESM, the sensing response of AuNP-functionalized sensors increases by increasing the relative humidity conditions. These results not only demonstrate the important role of $H_2O$ in the sensing mechanism but reaffirm the potential application of our sensing platform to be used for the detection of low $H_2S$ concentrations in exhaled breath where even higher humidity is expected.

Regarding $NH_3$, as expected, both AuNP-functionalized and non-functionalized sensors react similarly to all tested concentrations (Figs. 2(b) and 2(d)). It has been previously reported that the Schottky barrier modulation by the attachment of $NH_3$ molecules at the nanotube-electrode interfaces is the main contribution to the resistance change in low-density CNT networks like our sensors [55]. However, the addition of “nano-Schottky barriers” by AuNPs functionalization does not lead to an increase in sensitivity in this case. Unlike $H_2S$, ammonia molecules are not expected to be strongly chemisorbed on gold surfaces showing only a small charge transfer [56]. Therefore, we hypothesize that even though $NH_3$ can decrease the work function of AuNP upon interaction, the effect on the overall resistance change is minimal and modulation at the nanotube-electrode area, present in both types of sensors, remains the leading sensing mechanism.

A slightly increased sensing response to both gases was exhibited by unmodified sensors with higher initial electrical resistances (11 kΩ), indicating a better quality of nanotube distribution after dielectrophoretic deposition (Figs. 4(a) and 4(b)). On the contrary, no large variations were observed among the narrow initial resistance range (1–3 kΩ) of the AuNP-modified ones, demonstrating the robustness of fabrication (Figs. 4(c) and 4(d)). The fact that their response is comparable makes the presence of multiple sensors in an array a self-validation mechanism.

With a mixed population of AuNP-functionalized and bare sc-SWCNTs sensors, both $H_2S$ and $NH_3$ could be potentially determined in breath samples, considering the response of the bare CNTs to ammonia at higher levels, as demonstrated in our previous work [23]. To further demonstrate the selectivity of such approach, interaction to low ppb concentrations of NO gas was investigated, as also found in breath [57]. For this, sensors were exposed to 60 ppb and 1 ppm of NO for 15 min in a dry $N_2$ atmosphere to prevent NO oxidation. As it can be seen in Figs. S4(b) and S4(c) in the ESM, the sensors do not react to 60 ppb whereas a decrease in relative resistance change is observed in all sensors upon exposure to 1 ppm. The p-doping nature of NO molecules results in the increasing of charge carriers (holes) amount and the lowering of the Fermi level [58]. Non-functionalized sensors exhibit a higher negative response compared to AuNP-functionalized ones. Previous studies have shown the opposite trend indicating the improved sensitivity of AuNP-decorated-CVD-grown CNTs to NO gas [59]. However, we hypothesize that in our experiment, where lower concentrations and shorter exposure times are tested, the resistance change modulation is mainly governed by the interaction of NO gas molecules with sc-SWCNTs walls and that AuNP might serve only as blocking sites reducing the sensitivity of functionalized sensors.

## 2.3 Mathematical modeling

Output characteristics of AuNP-functionalized sensors presented higher $I_{SD}$ when compared to non-functionalized devices. However, the electrical behavior of both types can be modeled as a Schottky diode by the following Eq. [60, 61]

$$I_{SD} = I_0\left(e^{\frac{V_{SD}}{D^* V_0}} - 1\right) \quad (2)$$

where $I_0$ and $V_0$ are maximum values when $V_{SD}$ is positive and minimum values when $V_{SD}$ is negative. A value of 1.4 is considered for $D$ in all modeled curves and is obtained by global optimization of the total error between modeled and measured

**Figure 4** Sensing response ($\Delta R/R_0$) vs. initial resistance value of ((a) and (b)) non-functionalized and ((c) and (d)) AuNP-functionalized sensors for all $H_2S$ and $NH_3$ tested concentrations.

data. Notice that this $D$ parameter is equivalent to the "$n$" ideality parameter in a diode. As it can be seen in Fig. 5(a), the modeled $I$–$V$ curves reliably fit the measured ones from AuNP-functionalized and non-functionalized devices. In addition, experimental measurements can be normalized by scaling the $I$–$V$ curves in order to obtain $|V_0| = 1$ and $|I_0| = 1$. Figure 5(b) shows the results of the normalized current versus normalized voltage. It is apparent that the behavior of the whole set of curves is very similar for the two types of gas sensors and can be modeled using Eq. (2).

In order to model the change in electrical resistance upon exposure to the target gas, we propose a non-linear semi-empirical relation between the concentration and the sensing response in the following way [67, 68]

$$S = S_0\left(\frac{C}{C_0}\right)^K \tag{3}$$

where the sensing response ($S$) is obtained by Eq. (1), $C_0$ is the saturation concentration and $S_0$ is the corresponding sensing response. For non-functionalized devices $K \approx 1/D$ while for AuNP-functionalized sensors the expression in Eq. (3) is linear ($K \approx 1$) in the range from 20 to 160 ppb (Fig. 2(c)) and shows a non-linear behavior ($K \approx 1/D$) when higher concentrations are considered (Fig. S3 in ESM). The modeled and experimental results of sensing response to $H_2S$ and $NH_3$ for the two sensor types are presented in Figs. 5(c) and 5(d), where accurate model solutions in all treated cases can be observed.

The change of sensing response can be written by taking the derivative of Eq. (3) as following

$$\frac{\mathrm{d}S}{\mathrm{d}t} = K\cdot\frac{S_0}{C_0^K}\cdot C^{K-1}\cdot\frac{\mathrm{d}C}{\mathrm{d}t} \tag{4}$$

hence,

$$\frac{\mathrm{d}S}{\mathrm{d}t} = K\cdot\frac{S_0}{C_0^K}\cdot C^K\cdot\frac{\mathrm{d}C/\mathrm{d}t}{C} \tag{5}$$

using the derivative rule of the natural logarithm, the last equation can be re-written as follows

$$\frac{\mathrm{d}S}{\mathrm{d}t} = K\cdot\frac{S_0}{C_0^K}\cdot C^K\cdot\frac{\mathrm{d}}{\mathrm{d}t}[\ln(C)] \tag{6}$$

As it can be seen in Eq. (6), $\mathrm{d}S/\mathrm{d}t$ depends on the polarity of $\mathrm{d}/\mathrm{d}t[\ln(C)]$. When $\mathrm{d}S/\mathrm{d}t < 0$, the concentration decreases and the change of the concentration is negative, while for $\mathrm{d}S/\mathrm{d}t > 0$ the concertation increases, and the change of the concentration is positive.

Using the approximating $\mathrm{d}S/\mathrm{d}t \approx \Delta S/\Delta t$, we can write Eq. (6) for an instantaneous concentration $C_\mathrm{m}$ value at a given $\Delta t$ time as following

$$\frac{\Delta S}{\Delta t} = K\cdot\frac{S_0}{C_0^K}\cdot C_\mathrm{m}^K\cdot\frac{\Delta\ln(C)}{\Delta t} \tag{7}$$

replacing Eq. (3) in the last equation will give the following

$$\frac{\Delta S}{\Delta t} = K\cdot S_\mathrm{m}\cdot\frac{\Delta\ln(C)}{\Delta t} \tag{8}$$

hence,

$$\Delta S = K\cdot S_\mathrm{m}\cdot[\ln(C_\mathrm{m}+\Delta C)-\ln(C_\mathrm{m})] \tag{9}$$

It should be noticed that the sensing response depends on the value and polarity of the change of concentration, $\Delta C$. Therefore, $\Delta S$ for each given concentration $C_\mathrm{m}$ value at $\Delta t$, either it increases or decreases, can be written as

$$\Delta S = K\cdot S_\mathrm{m}\cdot\ln\left(1+\frac{\Delta C}{C_\mathrm{m}}\right) \tag{10}$$

or can be written as follows

$$\Delta S = \ln\left(1+\frac{\Delta C}{C_\mathrm{m}}\right)^{k\cdot S_\mathrm{m}} \tag{11}$$

Notice that this equation can be also used to describe the behavior of $\Delta C$ for each $\Delta t$ as following

**Figure 5** Mathematical modeling of electrical characteristics and sensing response. (a) Modeled (red lines) using Eq. (2) and measured output characteristics of non-functionalized and AuNP-functionalized sensors. (b) Normalized $I_{SD}$ vs. normalized $V_{SD}$ for all sensing devices. (c) Experimental (symbols) and modeled (lines) average sensing response ($\Delta R/R_0$) vs. target gas concentration for (c) AuNP-functionalized and (d) non-functionalized sensors. Experimental data are the same as presented in Fig. 2. Error bars are omitted for clarity.

$$\Delta C = C_m(e^{\frac{\Delta S}{K \cdot S_m}} - 1) \quad (12)$$

The last expression implies that $\Delta C$ converges exponentially to zero when $S_m$ approaches $S_0$, then the sensor is saturated and not able to sense the change in concentration.

## 3 Conclusion

We have successfully demonstrated the reproducible fabrication of a multiplexed sc-SWCNTs-based chemiresistive gas sensing platform for the selective detection of ppb concentrations of $H_2S$ in humid air at room temperature. Sensors functionalized with optimized size and distribution of AuNP showed a linear response in the range of 20–160 ppb with high reproducibility. Moreover, a low cross-sensitivity to relevant breath concentrations of $NH_3$ and NO was successfully demonstrated experimentally and described by a mathematical model. The reliability of multiple sensors in a single chip allows self-validation of the results to avoid false positives. The high sensitivity and selectivity of our sensors along with their operation at room temperature and low power consumption suggest their application for breath air analysis. A combined approach with multiple selective sensors toward $H_2S$ and unmodified ones for $NH_3$, together with the known opposite response for NO and the predictive information given by their modeling, can be seen as an important step forward in the development of self-validating diagnostic platforms analyzing multiple gases from breath. Although the self-validation approach can be achieved by implementing less sensors, we predict that the presence of such a high number of channels will play important roles in various aspects: (i) as resilience factor to face occasions of malfunction in some of them, (ii) space for integration of multiple materials and their functionalization for multiple gas detection purposes, and (iii) enabling deeper mathematical statistics and signal processing approaches such as cellular nonlinear networks.

## 4 Experimental

### 4.1 Electrode fabrication

Metal electrodes were fabricated on 4” silicon wafers with 500 nm of thermally grown $SiO_2$ layer by standard UV lithography process followed by the deposition of 3 nm of Cr and 50 nm of Au using metal evaporation and lift-off processes. Individual multichannel devices were then cut using a dicing machine (ESEC Dicing Saw 8003, DISCO, Kirchheim, Germany). Each device consisted of 64 gas sensors grouped in 4 quadrants of 16 sensors each with a common source/working electrode. Individual sensors presented an interdigitated electrode (IDE) area of 0.25 $mm^2$ with a finger width of 5 μm and a gap size of 2 μm. The process was potentially compatible with wafer-scale fabrication.

### 4.2 sc-SWCNTs dispersion

Purified 98% sc-SWCNTs with diameters of 1.2 to 1.7 nm and lengths up to 4 μm were purchased from Sigma-Aldrich (Darmstadt, Germany). 0.2 mg was dispersed in 12.5 mL of NMP by tip sonication for 2 h at 30% total power (Branson Digital Sonifier SFX 550, EMERSON, Missouri, USA) in an ice bath. The stable dispersion was then diluted 1:4 in NMP and re-sonicated for 10 min at the same conditions. Finally, it was centrifuged for 30 min at 15,000 rpm (Universal 320, Hettich, Tuttlingen, Germany) to remove nanotube agglomerations and titanium oxide particles, which can be generated during long sonication process. The supernatant was immediately used for the controlled dielectrophoretic deposition process.

### 4.3 Controlled dielectrophoretic deposition of sc-SWCNTs

The bare sensor chip was placed inside the test socket (Yamaichi) and fixed using two rubber rings to ensure correct electrical connections and proper sealing. 200 μL of the sc-SWCNT/NMP

dispersion was placed on the central part of the chip, covering the IDE areas, and an AC electric field of 8 $V_{pp}$ and 5 kHz was applied by a function generator (AgG 1022F, OWON Technology, The Netherlands) sequentially to each channel (individual gas sensor IDE structure) while monitoring the electrical resistance change of the channel using a custom Labview program (National Instruments, Texas, USA). After the deposition process, the devices were carefully cleaned with deionized (DI) water and baked for 10 min at 100 °C on a hotplate to evaporate residual NMP. More details can be found in the ESM.

### 4.4 Electrochemical deposition of AuNP

To assess the selective detection of low $H_2S$ concentrations in a humidified environment, we exploited the high chemical affinity of sulfur and gold by selectively functionalizing sc-SWCNTs with AuNPs. By applying the desired constant potential, current flows through the sc-SWCNTs, and metal cations in the electrolyte were then reduced and deposited on the nanotube walls [69].

AuNPs were electrodeposited on sc-SWCNTs using a three-electrode potentiostatic method (Fig. 1(e)). The common metal electrode connecting 16 sensors was used as the working electrode while a platinum and an Ag/AgCl wire were used as counter and reference electrodes, respectively. 1 mM $KAuCl_4$ solution in 100 mM KCl was used as the electrolyte. First, 200 μL was placed on the multichannel device covering one quadrant at a time (16 sensors). Then, counter and reference electrodes were immersed in the electrolyte and a constant potential of −0.4 V was applied for 30 s using a potentiostat (PalmSense4, Palmsense, Houten, The Netherlands). The applied voltage and duration were the results of an optimization process that can be found in the ESM. After the deposition process, the chip was rinsed with DI water and dried using nitrogen. A total number of 32 sensors were modified, leaving 32 unmodified ones as control. By keeping both sets of sensors on the same chip, we ensured the exact same conditions during the measurements.

### 4.5 Gas sensor characterization

The quality and distribution of sc-SWCNTs between metal electrodes and the electrodeposited AuNP were investigated using low-voltage scanning electron microscopy (LV-SEM) utilizing secondary/back-scattered electron detectors (Gemini SEM 500, ZEISS, Jena, Germany). The electrical characteristics of the gas sensing devices were obtained by measuring the $I$–$V$ curves sweeping the $V_{SD}$ from −2 to 2 V at room temperature using a source meter (2604B SourceMeter, Keithley Instruments, Germering, Germany).

### 4.6 Gas sensing experiments

To study the sensing properties, the fabricated devices were tested in a chemiresistive manner inside a self-made gas chamber system. The change in electrical resistance of the 64 sensors at a fixed $V_{SD}$ of 100 mV was measured by a custom-built multiplexing platform and a sourcemeter (2602 SourceMeter, Keithley Instruments, Germering, Germany). A membrane vacuum pump (412522, WELCH, Fürstenfeldbruck, Germany) was utilized to introduce a carrier airflow of 6 L/min inside the chamber and the precise addition of analyte gas was realized by mass flow controllers (Alicat Scientific Instruments, Duiven, The Netherlands). Before analyte gas exposure, the sensors were exposed to a constant airflow for 2 h to obtain a stable baseline. The temperature and relative humidity, with values of around 27 °C and 25%, were continuously monitored by a BME680 sensor (BOSCH, Ispringen, Germany) during the entire experiment and the sensing performance was directly compared to the commercially available H2S-B4 EC-sensor (Alphasense, Essex, UK). To address the comparative analysis of sensing response to $H_2S$ and NO, pure dry nitrogen was used as carrier gas instead of humid air with a total flow rate of 2 L/min.

## Acknowledgements

This work has been funded by the German Federal State of Saxony as part of the "SNIFFBOT: Sniffing Dangerous Gases with Immersive Robots" project under grant agreement number 100369691 and by the German Federal Ministry of Education and Research (No. 031B0298). Part of this work was conducted in the Dresden Center for Nanoanalysis (DCN) at the Center for Advancing Electronics Dresden (cfaed), TU Dresden.

**Funding note**: Open Access funding enabled and organized by Projekt DEAL.

**Electronic Supplementary Material**: Supplementary material (details on the dielectrophoretic deposition, AuNP functionalization optimization, full range of experimental and model $H_2S$ sensing response up to 820 ppb, and sensing response to NO gas) is available in the online version of this article at https://doi.org/10.1007/s12274-021-3771-7.

**Open Access** This article is licensed under a Creative Commons Attribution 4.0 International License, which permits use, sharing, adaptation, distribution and reproduction in any medium or format, as long as you give appropriate credit to the original author(s) and the source, provide a link to the Creative Commons licence, and indicate if changes were made.

The images or other third party material in this article are included in the article's Creative Commons licence, unless indicated otherwise in a credit line to the material. If material is not included in the article's Creative Commons licence and your intended use is not permitted by statutory regulation or exceeds the permitted use, you will need to obtain permission directly from the copyright holder.

To view a copy of this licence, visit http://creativecommons.org/licenses/by/4.0/.

## Electronic Supplementary Material

Selective and self-validating breath-level detection of hydrogen sulfide in humid air by gold nanoparticle-functionalized nanotube arrays
