# Supplementary material for: Selective and self-validating breath-level detection of hydrogen sulfide in humid air by gold nanoparticle-functionalized nanotube arrays
Source: Nano Res. 2021 Sep 2;15(3):2512–21. doi: 10.1007/s12274-021-3771-7 (PMC8412394; doi:10.1007/s12274-021-3771-7)
Supplement: Supplementary file 1 — Selective and self-validating breath-level detection of hydrogen sulfide in humid air by gold nanoparticle-functionalized nanotube arrays [file 12274_2021_3771_MOESM1_ESM.pdf]

Electronic Supplementary Material

Selective and self-validating breath-level detection of hydrogen sulfide in humid air by gold nanoparticle-functionalized nanotube arrays

Luis Antonio Panes-Ruiz[1,§], Leif Riemenschneider[1,§], Mohamad Moner Al Chawa[2,§], Markus Löffler[3], Bernd Rellinghaus[3], Ronald Tetzlaff[2], Viktor Bezugly[1,4,5] (✉), Bergoi Ibarlucea[1,5] (✉), and Gianaurelio Cuniberti[1,5] (✉)

[1] *Institute for Materials Science, Max Bergmann Center of Biomaterials, Technische Universität Dresden, Dresden 01062, Germany*
[2] *Institute of Circuits and Systems, Technische Universität Dresden, Dresden 01062, Germany*
[3] *Dresden Center for Nanoanalysis (DCN), Center for Advancing Electronics Dresden (cfaed), Technische Universität Dresden, Dresden 01062, Germany*
[4] *Life Science Incubator Sachsen GmbH & Co. KG, Dresden 01307, Germany*
[5] *Center for Advancing Electronics Dresden (cfaed), Technische Universität Dresden, Dresden 01062, Germany*
[§] *Luis Antonio Panes-Ruiz, Leif Riemenschneider, and Mohamad Moner Al Chawa contributed equally to this work.*

Supporting information to https://doi.org/10.1007/s12274-021-3771-7

Controlled dielectrophoretic deposition of sc-SWCNTs

After the sc-SWCNTs/NMP dispersion was placed on the IDE area of the multichannel device, a sinusoidal AC voltage (8 V_{pp}, 5 kHz) was applied by a function generator AgG 1022F (OWON Technology, The Netherlands) to the 64-channel multiplexer to assess one sensor at the time. Upon the formation of the sc-SWCNT network, the increasing electrical current was measured by an operational amplifier in a transimpedance configuration (TIA). The output of the TIA, a sinusoidal voltage proportional to this current, was then measured with a benchtop multimeter (34461a, Keysight Technologies, Boeblingen, Germany) and the electrical resistance was calculated and plotted. The process was stopped at a final resistance of 14 kΩ for each sensor (Figure S1) in order to obtain a reproducible set of sensors.

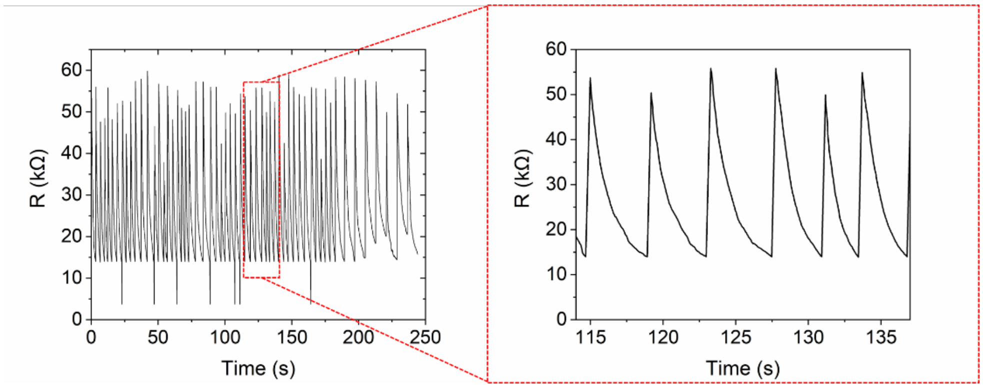

Figure S1 Continuous monitoring of electrical resistance during dielectrophoretic deposition of sc-SWCNTs on a 64-sensor device. The slower resistance decrease at the end of the process might indicate the long-term instability of the sc-SWCNT/NMP dispersion along with the small unavoidable defects at the metallic electrodes.

Address correspondence to Viktor Bezugly, v.bezugly@life-science-inkubator.de; Bergoi Ibarlucea, bergoi.ibarlucea@tu-dresden.de; Gianaurelio Cuniberti, gianaurelio.cuniberti@tu-dresden.de

TSINGHUA UNIVERSITY PRESS Springer

AuNP Functionalization Optimization

Three different deposition conditions were compared, by functionalizing three groups of 16 individual sensors each, in a single chip, as stated in Fig. S2(a). Two of the sets underwent an electrodeposition process at -0.4 V, for 30 and 60 s respectively. For the third one, a more negative and longer time was used, with the aim of obtaining a higher amount of larger particles. A fourth set was left unmodified. Afterward, the sensors were electrically characterized by measuring the output characteristics using a source meter (2604B SourceMeter, Keithley Instruments, Germering, Germany) and the distribution and size of nucleated AuNPs were analyzed by scanning electron microscopy (Gemini SEM 500, ZEISS, Jena, Germany).

Potentiostatic AuNP deposition showed the expected increase in current over time followed by the formation of a plateau, demonstrating the metal ion reduction and deposition on sc-SWCNTs walls [1–3]. Moreover, a more negative starting current value was measured for sensors treated at -0.8 V compared to -0.4 V. The difference between the two current curves obtained at the same potential of -0.4 V (green and blue in Fig. S2(b)) might come from variations in sensor resistance distribution or positioning errors during device contacting.

SEM characterization showed only a slight increase in average particle diameter from 60 nm to 80 nm by increasing deposition time and voltage magnitude. Nevertheless, a lower number of AuNP and larger separation distances (>100 nm) between nucleation sites were observed for deposition at -0.4 V for 30 s, whereas a continuous coating along the sc-SWCNTs due to the contact of neighboring nucleated particles was more likely to occur in sensors treated for a longer time and at more negative voltages (Figure S2(d)-(f)). The results correlate to the measured *IV* curves where a systematic increase of I_{SD} by increasing the deposition time and applied voltage magnitude was observed. Most of the sensors subjected to 1 min deposition at -0.8 V showed a linear ohmic curve indicating that the electrical conduction was through the continuous gold coating as observed in the SEM characterization. Interestingly, our results showed an opposite trend compared to *IV* curves reported by Mubeen *et al* [4] after AuNP deposition. The difference might come from the type of nanotubes used as starting material. In their case, carboxylated-SWCNTs with 80-90% purity might have experienced de-doping during the AuNPs deposition process, which would explain the decrease in I_{SD}.

Based on these findings, a voltage of -0.4 V and a deposition time of 30 s were selected for device fabrication presented in the main text.

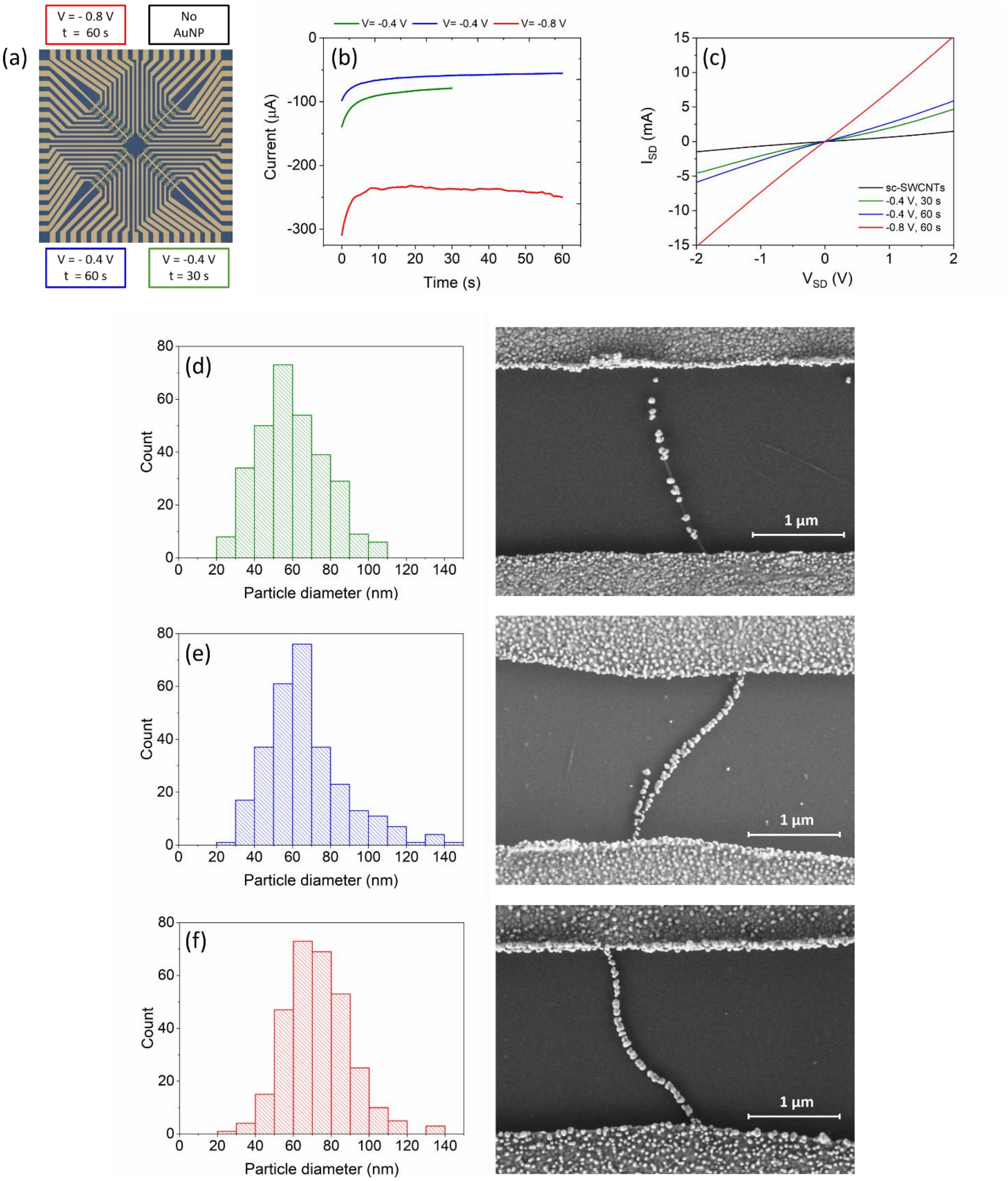

Figure S2 Optimization of AuNP functionalization. (a) Voltage and deposition times used for each group of 16 sensors in a multichannel device. (b) Measured current during potentiostatic AuNP deposition for each sensor group. (c) *IV* curves of sensors after AuNP deposition. Histograms and SEM images of individual agglomerations of sc-SWCNTs after AuNP electrodeposition for (d) 30 s and (e) 60 s at -0.4 V and (f) -0.8 V for 60 s.

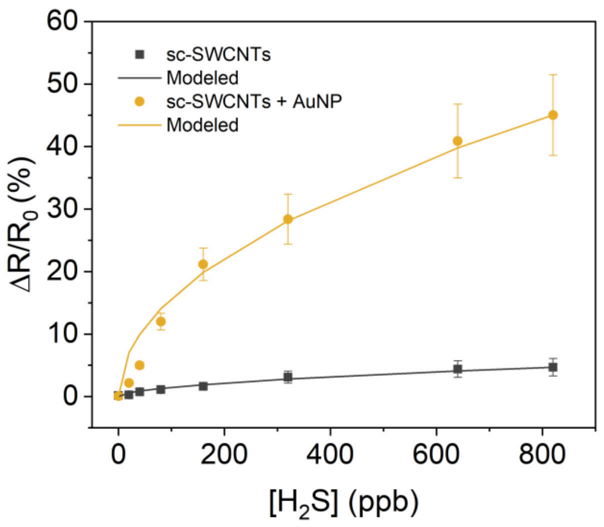

Figure S3 Measured (symbols) and modeled (lines) sensing response of AuNP-functionalized and non-functionalized sensors to 20, 40, 80, 160, 320, 640 and 820 ppb of H_2S.

NO gas cross-sensitivity

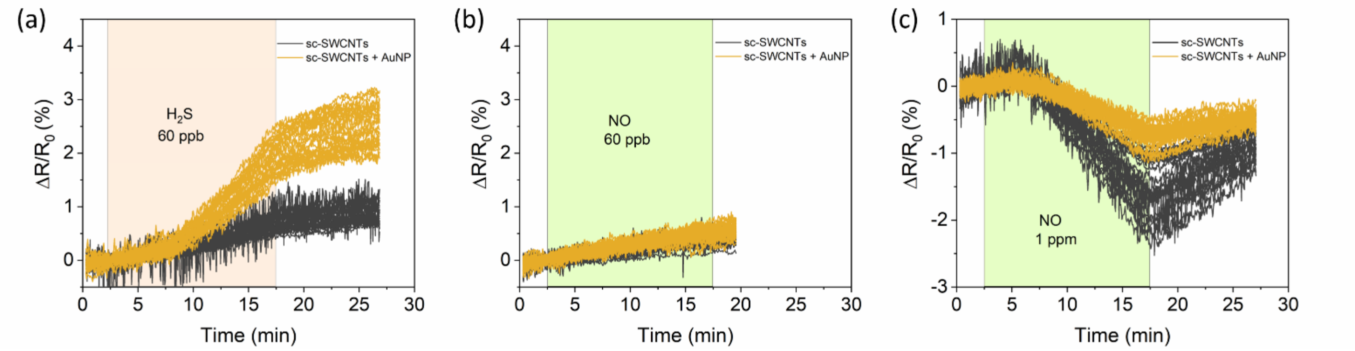

Figure S4 Sensing response (*ΔR/R₀*) of AuNP-functionalized and non-functionalized sensors to 60 ppb of (a) H_2S and (b) 60 ppb, (c) 1 ppm of NO gas in a dry nitrogen atmosphere.

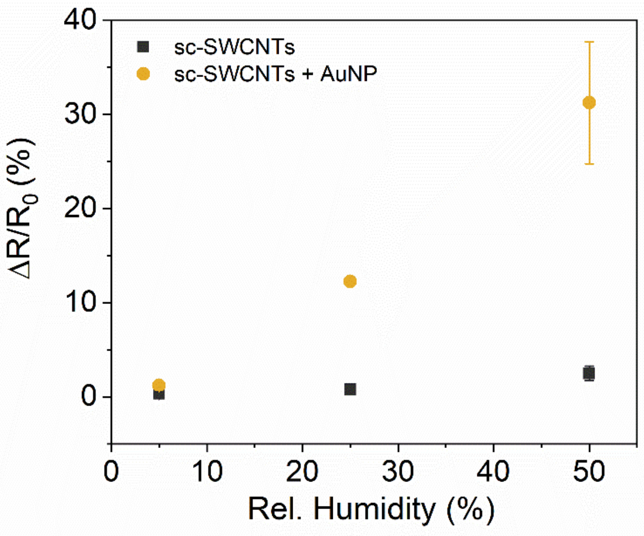

Figure S5 Sensing response of AuNP-functionalized and non-functionalized sensors to 100 ppb of H_2S at 5%, 25% and 50% relative humidity. Values plotted at 25% relative humidity were calculated using the mathematical model presented in the main text.

References

[1] Choi, H. C; Shim, M.; Bangsaruntip, S.; Dai H. Spontaneous reduction of metal ions on the sidewalls of carbon nanotubes. *J. Am. Chem. Soc*. **2002**, *124*, 9058-9059.

[2] Quinn, B. M.; Dekker, C.; Lemay, S.G. Electrodeposition of noble metal nanoparticles on carbon nanotubes. *J. Am. Chem. Soc*. **2005**, *127*, 6146-6147.

[3] Kim, D. S.; Lee, T.; Geckeler, K. E. Hole-doped single-walled carbon nanotubes: Ornamenting with gold nanoparticles in water. *Angew. Chemie* **2006**, *45*, 104-107.

[4] Mubeen, S.; Zhang, T.; Chartuprayoon, N.; Rheem, Y.; Mulchandani, A.; Myung N. V.; Deshusses, M. A. Sensitive detection of H2S using gold nanoparticles decorated single-walled carbon nanotubes. *Anal. Chem*. **2010**, *82*, 250-257.

Address correspondence to v.bezugly@life-science-inkubator.de, bergoi.ibarlucea@tu-dresden.de, gianaurelio.cuniberti@tu-dresden.de
